# Effects of a Dance-Based Intervention on Affective States and Self-Esteem in Adolescents Receiving Psychiatric Care: Differences Between Youth with and Without Problematic Internet Use—A Pilot Study

**DOI:** 10.3390/bs16020170

**Published:** 2026-01-26

**Authors:** Sarah Al Schameri, Belinda Plattner, Lucas Rainer, Helena Gampe, Bernhard Salcher, Katarzyna Grebosz-Haring, Marie-Christine Klettner, Kornelius Winds

**Affiliations:** 1 Department of Child and Adolescent Psychiatry and Psychotherapeutic Medicine, Salzburger Landeskliniken, Paracelsus Medical University Salzburg, 5020 Salzburg, Austria; sarah.al-schameri@stud.pmu.ac.at (S.A.S.); belinda.plattner@teach.pmu.ac.at (B.P.); lucas.rainer@teach.pmu.ac.at (L.R.); h.gampe@salk.at (H.G.); marie.christine.klettner@sbg.at (M.-C.K.); 2 Centre for Cognitive Neuroscience Salzburg, University of Salzburg, 5020 Salzburg, Austria; 3 Department of Neurology, Neurocritical Care and Neurorehabilitation, Salzburger Landeskliniken, Member of the European Reference Network, EpiCARE, Paracelsus Medical University Salzburg, 5020 Salzburg, Austria; 4 Department of Environment and Biodiversity, University of Salzburg, 5020 Salzburg, Austria; bernhard.salcher@plus.ac.at; 5 Interuniversity Organisation Arts & Knowledges, Mozarteum University Salzburg, 5020 Salzburg, Austria; katarzyna.grebosz-haring@plus.ac.at; 6 Department of Art History, Musicology and Dance Studies, University of Salzburg, 5020 Salzburg, Austria

**Keywords:** problematic internet use, child and adolescent psychiatry, dance, self-esteem, emotionality

## Abstract

Problematic internet use (PIU) in adolescence is associated with adverse psychological outcomes, including emotional symptoms and impaired self-perception. *The influence of PIU* on physical activities such as dancing remains unclear. This study examined the psychological effects of a dance intervention in a psychiatry setting, focusing on changes in self-esteem and positive and negative emotions comparing adolescents with (PIU+) and without (PIU−) PIU. Eighteen adolescents (mean age = 15.39 years; 10 female) participated in four weekly dance workshops (WSs). Assessments used measures of self-esteem, affect, PIU, and psychiatric diagnosis. Of the sample, 44.4% met criteria for PIU. At baseline (BL), PIU+ adolescents had significantly lower self-esteem (*p* = 0.018) and higher internalizing disorders (*p* = 0.041). PIU+ showed a trend toward reduced negative emotion between BL and WS3 (*p* = 0.063) and significant self-esteem increases from BL to WS2 (*p* = 0.043) and WS3 (*p* = 0.042). In PIU−, positive and negative emotion decreased from BL to WS1 (*p* = 0.008; *p* = 0.007), while negative emotions increased from WS1 to WS2 (*p* = 0.027). These findings indicate longitudinal effects of PIU on emotional functioning. Dance interventions may reduce negative emotion and improve self-esteem, supporting use as a clinical treatment approach for adolescents with PIU.

## 1. Introduction

Childhood and adolescence constitute key developmental phases during which emotional regulation, self-concept, and social competence are established, providing the foundation for long-term mental health (Fombouchet et al., 2023; Gómez-López et al., 2022; Tolstrup et al., 2025). During these years, young people encounter a range of stressors, including social comparison, peer pressure, and identity exploration, which may increase vulnerability to psychological difficulties when coping resources are insufficient (Compas et al., 2017). Within adolescent development of self-esteem constitutes a key psychological concept that encompasses global attitudes (Maslow, 1954; Rosenberg, 2006; Rosenberg et al., 1995), domain-specific evaluations (Cast & Burke, 2002; Harter, 2012), cognitive self-evaluations (Ek et al., 2008) and affective dimensions of ‘self-liking’ (Brown & Marshall, 2006). During puberty, adolescents, particularly girls, often experience increased body dissatisfaction and emotional volatility, which directly impact self-esteem (Moksnes & Reidunsdatter, 2019; Paxton et al., 2006). Recent evidence suggests that girls may exhibit a progressively stronger vulnerability effect over time (Gao et al., 2022; Sowislo & Orth, 2013).

Problematic internet use (PIU) has emerged as a particularly relevant factor influencing emotional and behavioral functioning in adolescents. PIU is characterized by excessive or poorly controlled online activity that leads to distress or functional impairment, and has been linked to depression, anxiety, attentional problems, and interpersonal conflict (Cai et al., 2023; Restrepo et al., 2020; Soriano-Molina et al., 2025). A large cross-sectional study of 11,956 adolescents from 11 European countries found that the prevalence of PIU was 4.4% (Durkee et al., 2012), with substantially higher rates reported in clinical populations (Fuchs et al., 2018; Winds et al., 2022). A more recent meta-analysis, which included 131 epidemiological studies from 31 countries involving over 690,000 participants, estimated the prevalence of generalized internet addiction to be 7.02% (Pan et al., 2020). Neuroimaging studies indicate that PIU is associated with altered connectivity in networks supporting executive control, emotion regulation, and interoceptive processes suggesting a neurobiological basis for observed behavioral difficulties (Darnai et al., 2019).

During adolescence, self-esteem constitutes a key determinant of mental health and appears closely linked to PIU, with accumulating evidence indicating complex bidirectional associations (Johnson et al., 2016; Lai et al., 2023; Rosenberg et al., 1989). The association between self-esteem, PIU, and depression has been conceptualized through two principal frameworks: the vulnerability model, which posits that low self-esteem precipitates psychological distress through maladaptive coping and rumination (Dumont & Provost, 1999; Sowislo & Orth, 2013), and the scar model, which suggests that symptoms of PIU and perceived loss of control progressively undermine self-concept over time (Zeigler-Hill, 2011). Recent research provides more nuanced insights by distinguishing stable traits from temporal fluctuations. Findings suggest that the salience of vulnerability versus scar effects may depend on the specific developmental stage (Gao et al., 2022; Orth et al., 2021).

Low self-esteem may undermine emotion regulation capacities, thereby increasing vulnerability to pathological usage patterns as a maladaptive coping strategy (Villanueva-Blasco et al., 2026). Self-esteem also shapes adolescents’ self-perception and their ability to meet social, academic, and emotional demands; consequently, low self-esteem has been associated with depression, anxiety, and reduced well-being (Orth & Robins, 2014). Notably, PIU has been linked to reduced self-esteem and emotional dysregulation, suggesting a reinforcing cycle of maladaptive online behavior (Villanueva-Blasco et al., 2026). Emotional functioning and self-esteem represent core developmental pillars in adolescence and constitute central therapeutic targets in psychiatric settings. Emotional dysregulation is a transdiagnostic characteristics across a wide range of disorders (Compas et al., 2017), whereas frequent positive emotional experiences foster resilience and support long-term psychosocial adjustment (Gilchrist et al., 2023). As untreated mental health problems in adolescence often persist into adulthood, early detection and developmentally sensitive interventions are essential (Tolstrup et al., 2025).

Conventional verbally oriented psychotherapies, although effective for many, may not sufficiently address the complex emotional and developmental needs of young patients. Adolescents often encounter difficulties articulating psychological distress or may disengage from traditional talk-based therapeutic formats (Bhide & Chakraborty, 2020). Consequently, clinicians increasingly emphasize integrative and multimodal interventions that align with adolescents’ lived experiences, foster agency, and incorporate nonverbal and embodied modes of expression (Svamo et al., 2024). In parallel, scientific consensus underscores the need for evidence-based approaches to address PIU (Mathew et al., 2020). Cognitive Behavioral Therapy (CBT) (Butler et al., 2006; Young, 2009) and Multifamily Therapy (Zhou & Wang, 2021) remain dominant, whereas Positive Psychology frameworks have introduced valuable strategies for enhancing social quality and positive emotions (Layous et al., 2011; Park et al., 2016).

Beyond established psychotherapeutic approaches, recent meta-analytic evidence indicates that school-based programs are highly effective in reducing PIU symptoms—far more than reducing screen time, particularly when they are therapy-based, externally led, and targeted at at-risk youth (Žmavc et al., 2025). Within this landscape, physical activity serves as a vital protective factor, mitigating PIU both directly and indirectly by fostering family cohesion and prosocial behavior (Zhu et al., 2025). Furthermore, mind–body interventions like mindfulness and tai chi significantly outperform placebos in symptom alleviation (Jia et al., 2025). Notably, mindfulness, yoga and dance sport have been identified as the most effective modalities for young adults, with dance categorized as a top-tier strategy. This reinforces the theoretical shift toward movement-based therapeutic options for managing PIU (Jia et al., 2025).

In psychiatric settings, the presence of PIU may complicate treatment. Adolescents with PIU tend to show greater symptom severity, lower self-esteem, and more emotional dysregulation than their non-PIU peers (Gioia et al., 2021). When inpatient programs restrict smartphone or internet access, a common safety measure, affected adolescents may exhibit withdrawal-like symptoms, irritability, or reduced engagement with treatment staff (Burke et al., 2022). These clinical challenges emphasize the need for interventions that help restore motivation, emotional regulation, and embodied presence in this subgroup.

Physical activity represents a promising complementary component of adolescent psychiatric care. Regular physical activity is associated with improved mood, reduced anxiety and depressive symptoms, and enhanced cognitive functioning among young people, whereas sedentary behavior—often linked to excessive screen time—is associated with poorer emotional well-being, decreased self-concept, and higher psychological distress (Rodriguez-Ayllon et al., 2019). Integrating physical activity intervention into psychiatric treatment may therefore mitigate some of the negative consequences associated with PIU and inactivity (Wang et al., 2025). Physical activity can further enhance positive affect through neurobiological mechanisms involving dopaminergic and serotonergic pathways (Lin & Kuo, 2013).

Within this context, dance offers a particularly compelling intervention for adolescents. Dance integrates physical activity, aesthetic and emotional experience, creativity, music and rhythm, and interpersonal synchrony, creating a multilayered therapeutic experience that engages both body and mind (Koch et al., 2019; Tao et al., 2022). Research consistently demonstrates that dance and dance movement therapy have positive effects on depression, anxiety, affect regulation, body awareness, and self-esteem (Anderson et al., 2014; Koch et al., 2019; Tao et al., 2022).

Findings from a study conducted with 7- to 14-year-old children and adolescents in outpatient residential care settings, show that the intervention’s quantitative effects were not homogeneous but varied across subgroups based on factors such as gender, migration background, and parental mental illness. Specifically, female participants showed a significant reduction in depressive symptoms, while male participants exhibited an increase in these symptoms. Additionally, children with mentally ill parents experienced a significant decrease in anger, while those with a migration background showed a worsening of self-worth, empathy, optimism, self-efficacy, and sense of coherence (Birnkammer & Calvano, 2023).

Although empirical evidence in clinical populations is currently lacking, adolescents presenting PIU—who often experience reduced embodied awareness and emotional dysregulation—may represent a subgroup that could be particularly responsive to dance- and movement-oriented interventions.

Consequently, interventions that engage affective and body-related self-evaluations—such as the dance movement therapy investigated in the present study—are clinically relevant in the treatment of adolescents, as they may disrupt the cycle of diminished self-control and negative self-perception. Dance-based approaches may address two central challenges observed among adolescents with PIU: emotional dysregulation and low self-esteem. By facilitating creative expression, social interaction, and the development of physical competence, dance allows adolescents to experience their bodies as agentic and expressive, countering patterns of passivity or avoidance. Emerging evidence further suggests dance- or movement-based interventions can improve interoception, self-perception, and emotional states, even over short intervention periods (Koch et al., 2019; Schwender et al., 2018).

Although movement-based therapies have shown promising effects in school settings, their impact on adolescents in acute psychiatric inpatient care—particularly those presenting with co-occurring PIU symptoms—remains insufficiently examined. The present pilot study sought to address this gap by evaluating a specialized dance intervention in a clinical adolescent population. We investigated whether adolescents with and without PIU symptoms displayed differential improvements in self-esteem and affective states. In line with previous research, we anticipated that participation in dance would enhance self-esteem and emotional well-being in the full sample, and we further explored potential group differences between PIU+ and PIU− youth.

## 2. Materials and Methods

### 2.1. Participants

In total, 35 children and adolescents undergoing therapeutic or rehabilitative treatment at the Department of Child and Adolescent Psychiatry and Psychotherapeutic Medicine, Salzburger Landeskliniken, Paracelsus Medical University Salzburg, Salzburg, Austria, met the inclusion criteria for the study and were invited to participate. Recruitment took place within therapeutic inpatient and day-clinic settings, where patients are typically treated for longer periods due to pronounced or chronic psychopathology. Of the eligible patients admitted during the study period, 18 agreed to participate, while 17 (8 females, 9 males) declined participation, resulting in a final sample of 18 participants.

Participants ranged in age from 11 to 18 years at the time of the study (M = 15.39, SD = 0.35). All participants were receiving treatment in either a day clinic or inpatient setting. Psychopathology was diagnosed clinically according to the International Statistical Classification of Diseases and Related Health Problems, 10th Revision (ICD-10). Comorbid psychiatric diagnoses were permitted. Clinical diagnoses, obtained from medical records included affective disorders (n = 3), neurotic, stress-related and somatoform disorders (n = 14), eating disorders (n = 2), personality disorders (n = 3), sexual maturation disorders (n = 1), developmental disorders (n = 1) and behavioral and emotional disorders with onset in childhood and adolescence (n = 2).

Inclusion criteria were: (1) age between 10 and 18 years; (2) current treatment in the day clinic or inpatient setting; (3) written informed consent provided by both the patient and their legal guardians. Exclusion criteria were: (1) insufficient reading or comprehension skills preventing completion of self-report measures; (2) acute suicidality or psychosis; (3) documented history of severe trauma; (4) acute phase of an eating disorder.

All participants underwent the same intervention procedure. The assignment into PIU− and PIU+ groups was performed post hoc for analytical purposes. Throughout the survey, missing data occurred due to incomplete completion of questionnaires. A detailed overview of missing data per variable and group (PIU− and PIU+) is provided in Table A1 in the Appendix A. Additional missing data resulted from non-attendance at workshop 3 and 4. At workshop 3, one participant from the PIU− group and one participant from the PIU+ group did not attend. At workshop 4, two participants from the PIU− group and two participants from the PIU+ group were absent.

### 2.2. Dance Intervention

Inpatient and day-clinic patients at our institution are offered a daily educational leisure program aimed at providing new experiences and helping adolescents discover recreational activities that they may continue after discharge. The dance intervention was embedded within this program and was therefore conceived as a leisure-based activity rather than a formal therapeutic treatment. Accordingly, the workshops (WS) were accompanied by educational staff and took place outside the clinical setting, at the dance studio of the University of Salzburg.

The workshops were led by the professional dancer Seraphim Schuchter, who studied contemporary dance and dance pedagogy in Vienna, Linz, and Barcelona, and who has experience working with children and adolescents with psychiatric disorders. Patients took part in four dance sessions, which were held once a week for one hour. Each consecutive workshop followed the same structured sequence. This approach fostered a sense of routine, which is particularly important when working with adolescents experiencing mental health difficulties.

Each of the four workshop-interventions consisted of two complementary components: In the first part of the workshop participants engaged in the repeated practice of structured dance sequences. Those were created as a fusion of dance technique material and movement exercises drawing from various styles of contemporary dance, Afro/HipHop, and Bowspring—a dynamic form of yoga based on the body’s natural curves. This continuous and targeted work on choreographic material was designed to establish routine, support skills development, and facilitate experiences of personal achievement.

At the end of each workshop, calming breathing exercises and guided meditations (including a body scan) were incorporated, aiming to support participants in developing a heightened internal perception of their own bodies and, consequently, greater mindfulness and emotional regulation.

### 2.3. Measures

#### 2.3.1. Problematic Internet Use (PIU)

PIU was assessed using the 14-item Compulsive Internet Use Scale (CIUS) (Meerkerk et al., 2009). Items are rated on a 5-point Likert scale, with a maximum total score of 56 points. A higher score indicates a higher level of PIU severity. The authors of the CIUS proposed a cut-off score of 28 points for compulsive internet use (Meerkerk et al., 2009; Rehbein et al., 2025; Rumpf et al., 2014). Other authors have proposed cut-off scores of ≥18 (with 79.7% sensitivity and 79.4% specificity) and >21 points for identifying PIU and estimating its prevalence (Guertler et al., 2014). The CIUS has been validated in representative samples of both heavy and regular internet users. The scale demonstrated good factorial stability across different samples and subsamples, as well as over time. The test exhibited high internal consistency (α = 0.89–0.90) and good validity (Meerkerk et al., 2009). The CIUS has been translated into several languages and is available in different formats, including original and shortened versions (Lopez-Fernandez et al., 2019; Rehbein et al., 2025). A representative sample of adolescents was used to validate the German version, and the results were consistent with those of the original Dutch version, demonstrating high internal consistency (α = 0.929) (Wartberg et al., 2014).

Instead of relying on total Internet usage time—which demonstrates only small-to-moderate associations with psychological and educational wellbeing (r = −0.14 to 0.33)—we employed the Compulsive Internet Use Scale (CIUS) (Sanders et al., 2024). The CIUS emphasizes compulsive engagement and loss of control, constructs regarded as more robust indicators of problematic Internet use than cumulative screen hours. We applied a cut-off score of 28 points to identify adolescents meeting criteria for PIU. Although PIU can be conceptually dimensional, we adopted a categorial classification to enhance clinical interpretability. Both the CIUS and the corresponding cut-off score are widely utilized in epidemiological and clinical research to differentiate non-problematic from problematic usage and provide a sensitive metric for detecting loss of control and psychosocial impairment in youth (Fuchs et al., 2018; Rehbein et al., 2025; Rumpf et al., 2014; Winds et al., 2022).

#### 2.3.2. Screening of Mental Health Symptoms

Mental health symptoms were screened using the screen self-rating scale of the German Diagnostic System for Mental Disorders, according to the ICD-10 and DSM-5 criteria for children and adolescents (in German: Diagnosesystem für psychische Störungen nach ICD-10 und DSM-5 für Kinder und Jugendliche) (DISYPS-III SBB-Screen) for adolescents aged 11–18 years (Döpfner & Görtz-Dorten, 2017). The instrument assesses symptoms from the most important areas of child and adolescent disorders, such as affective, attentional, anxiety-related, obsessive-compulsive, social communication-related, trauma- and stress-related, tic-related, behavioral, as well as attachment- and relationship-related domains. Items are rated for symptom frequency over the past four weeks. The DISYPS-III is a psychometrically robust diagnostic system, with acceptable to good internal consistency across its various symptom scales (α = 0.70–0.90) (Döpfner & Görtz-Dorten, 2017).

#### 2.3.3. Self-Esteem

Self-esteem was assessed using the Self-esteem inventory for children and adolescents (German: Selbstwertinventar für Kinder und Jugendliche) (SEKJ), designed for children and adolescents (Schöne & Stiensmeier, 2016). Items are rated on a 5-point Likert scale, consisting of 32 items in total, based on either agreement or disagreement. The SEKJ was standardized using a representative sample of children and adolescents ranging in age from 10 to 16 years. It demonstrated good to excellent internal consistency across all three self-esteem domains (α = 0.81–0.86 for 10–12-year-olds and α = 0.87–0.90 for 13–16-year-olds) and a high retest-reliability (Schöne & Stiensmeier, 2016).

#### 2.3.4. Affective States

Affective states were measured using the Positive and Negative Affect Schedule (PANAS) (Watson et al., 1988). The PANAS is a widely used self-rating instrument comprising 20 adjectives relating to specific sensations and feelings, which are rated on a 5-point Likert scale. It assesses positive and negative state emotions, summarized based on 10 items respectively (Breyer & Bluemke, 2016; Watson et al., 1988). The scale shows a high internal consistency reliability (α = 0.86–0.90 for positive emotions and α = 0.84–0.87 for negative emotions) and stability over (Watson et al., 1988). The original English version has been validated in German, resulting in psychometric properties that satisfy the scale’s requirements (Krohne et al., 1996).

#### 2.3.5. Narrative Feedback

In narrative feedback participants were asked to provide responses addressing the following questions: “What thoughts and physical impressions did you have during the workshop?” and “How did you feel during the dance exercises?”.

### 2.4. Procedure

The weekly dance workshops were held between April 2023 and May 2023. Recruitment at the clinic took place two weeks prior to the workshop. Medical staff introduced the project during individual conversations, verify the inclusion and exclusion criteria, explained the study procedures, and obtained written informed consent.

One week prior to the dance workshop, baseline assessments (BL) were conducted, including the CIUS, DISYPS SBB-Screen, SEKJ and PANAS. After each WS participants completed the PANAS and SEKJ and provided brief narrative feedback. The tests and narrative feedback were entered and evaluated after the dance workshops had been completed, meaning that the dance instructor and study staff were blind to the grouping variable relating to PIU; group allocation was carried out retrospectively as part of the statistical analysis.

### 2.5. Statistical Analysis

Given the exploratory nature of this pilot study and the limited sample size, non-parametric Friedman tests were used to examine within-subject changes in positive affect, negative affect, and self-esteem across measurement points: baseline (BL), workshop 1 (WS1), workshop 2 (WS2), workshop 3 (WS3) and workshop 4 (WS4). No adjustments for multiple comparisons were applied; findings should therefore be interpreted as hypothesis-generating. Post hoc analyses were conducted using Wilcoxon signed-rank tests to compare baseline with each workshop (BL–WS1, BL–WS2, BL–WS3, BL–WS4) as well as adjacent workshops (WS1–WS2, WS2–WS3, WS3–WS4) to characterize the temporal progression of effects.

Statistical analyses were conducted in SPSS version 29 (IBM Corp., 2023). Participants were divided post hoc into PIU+ and PIU− groups using a CIUS cut-off score of 28 points to identify problematic Internet use (Meerkerk et al., 2009; Rehbein et al., 2025). Missing data across the repeated measurements (WS1–WS4) were handled using available case analysis. Group differences in demographic characteristics, Internet usage time (CIUS), internalizing and externalizing symptoms (DISYPS-III), and BL measures of self-esteem (SEKJ) and positive and negative emotion (PANAS) were examined using Fisher’s exact tests for categorial variables and Mann–Whitney U-tests for continuous variables.

Effect sizes for categorial comparisons were calculated using Cramer’s V (small > 0.10, medium > 0.30, large > 0.50) (Cohen, 1988). For non-parametric continuous comparisons, effect sizes were expressed as r (r = Z/$\sqrt{N}$) using the same interpretive thresholds (Cohen, 1988). Given the exploratory nature of this pilot study and the limited sample size, non-parametric Friedman tests were used to examine within-subject changes in positive emotions, negative emotions, and self-esteem across measurement points (BL, WS1, WS2, WS3, WS4). No adjustments for multiple comparisons were applied; findings should therefore be interpreted as hypothesis-generating. Post hoc analyses were conducted using Wilcoxon signed-rank tests to compare BL and each WS (BL–WS1, BL–WS2, BL–WS3, BL–WS4), as well as adjacent WS (e.g., WS1–WS2, WS2–WS3, WS3–WS4) to characterize the temporal progression of effects.

Narrative feedback was analyzed using qualitative content analysis according to Mayring (Mayring, 2023). Due to the exploratory nature of the study, categories were generated inductively from the data. All narrative responses to the question “What thoughts and physical impressions did you have during the workshop?” were included in the analysis and semantically related statements were summarized into content-based categories. The analysis was conducted at a descriptive level, with the aim of identifying recurrent themes in participants’ experiences and enriching the quantitative findings. Category frequencies were calculated to provide an overview of the distribution of themes across workshops and groups. These frequencies are presented visually using word clouds generated with World Cloud Generator provided by ETH Zürich https://wordclouds.ethz.ch/ (accessed on 3 January 2025) (World Cloud Generator, n.d.). Word clouds were used for illustrative purposes only and should be interpreted descriptively. For comparative display, word clouds were generated separately for the PIU− and PIU+ groups.

### 2.6. Study Ethics

The study was conducted in accordance with the Declaration of Helsinki 1995 (as revised in Edinburgh in 2000) and approved by the Ethics Committee on the 26 April 2023 (ethics committee vote number: 1054/2023) of the state of Salzburg. All participants and their legal custodians provided written informed consent before participating in the study.

## 3. Results

### 3.1. Descriptive Measures/Demographics

Due to the exploratory nature of this pilot study and the limited sample size (n = 18), all statistical findings presented below should be interpreted as hypothesis-generating observations rather than confirmatory evidence. The study population consisted of 18 patients with a mean age of 15.39 (SD = 1.50), of whom ten (55.6%) were female and eight (44.4%) were male. Ten patients (55.6%) received in-patient treatment, and eight (44.4%) received day-clinic treatment. Based on the Compulsive Internet Use Scale (CIUS) cut-off score at 28 points, 8 patients (44.4%) were classified as exhibiting PIU+, while 10 (55.6%) did not (PIU−). Regarding gender 4 girls (40%) and 4 boys (50%) met the criteria for PIU. The mean daily internet use time was 6.28 h/day (SD = 3.79) on weekdays and 8.1 h/day (SD = 5.45) on weekends. No significant difference in average internet use time was found between PIU+ and PIU− (see Table 1). There were no significant differences regarding clinical diagnosis in between the PIU+ and PIU− group.

#### Descriptives in Relation with PIU

The mean baseline self-esteem score (SEKJ) for the total sample was 24.61 (SD = 10.15). The PIU+ subgroup showed significantly lower self-esteem at baseline than the PIU− subgroup (*p* = 0.018) (see Table 1). Seven out of eight participants in the PIU+ subgroup had a self-esteem score below the norm, which was significantly higher than in the PIU− subgroup (*p* = 0.025) (see Table 1). No participant scored above the normative range for self-esteem. Internalizing symptoms were significantly more prevalent in the PIU+ subgroup (*p* = 0.041). Furthermore, pathological internalizing symptoms were found in all PIU+ subgroup participants (see Table 1). A Mann–Whitney U test was performed to compare the means of positive and negative emotions and self-esteem from BL and each WS between the PIU subgroups. Except for self-esteem at BL (z = −2.361, *p* = 0.018) and for negative emotions at WS 1 (z = −1.997, *p* = 0.046), no significant differences could be detected for between the PIU+ and PIU− groups.

### 3.2. The Course of Positive Emotions, Negative Emotions, and Self-Esteem in the Course of the Intervention

**Figure 1:**
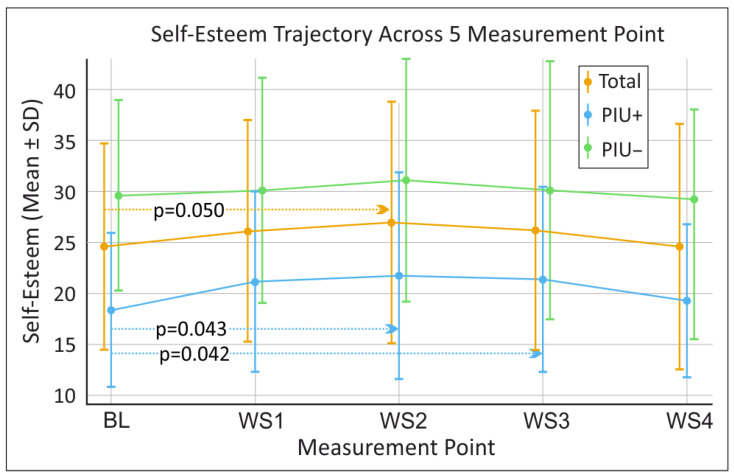
Longitudinal mean findings for self-esteem as measured by the Self-Esteem Inventory for Children and Adolescents (SEKJ) over time for the total sample as well as for participants with and without problematic internet use (PIU+ and PIU−, respectively). BL = baseline, WS1 = workshop 1, WS2 = workshop 2, WS3 = workshop 3, WS4 = workshop 4. Horizontal dotted arrows indicate the time-point comparisons evaluated via Wilcoxon post hoc tests; for clarity, only significant results and trends are illustrated. Significant results and trends are presented with their corresponding *p*-values, which were derived from Wilcoxon post hoc tests performed to evaluate differences in changes in scores between the measurement points. Sample size for individual time points may vary slightly due to workshop attendance and data completeness (Available case analysis: BL n = 18; WS1 n = 18; WS2 n = 18; WS3 n = 18; WS4 n = 15).

**Figure 2:**
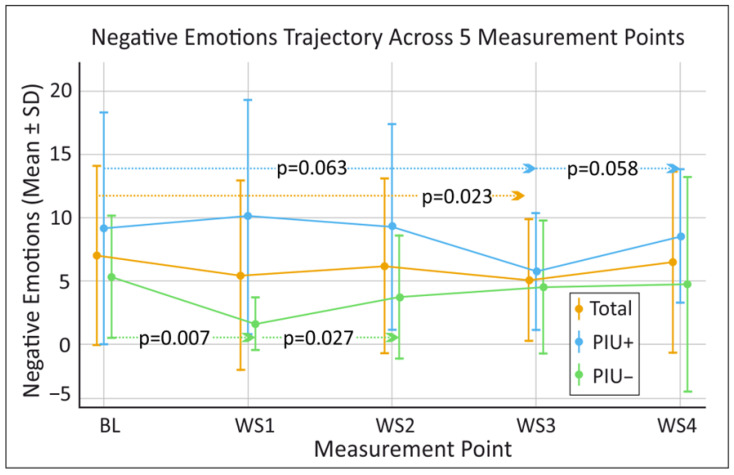
Longitudinal mean findings for negative emotions as measured by the Positive and Negative Affect Schedule (PANAS) over time for the total sample as well as for participants with and without problematic internet use (PIU+ and PIU−, respectively). BL = baseline, WS1 = workshop 1, WS2 = workshop 2, WS3 = workshop 3, WS4 = workshop 4. Horizontal dotted arrows indicate the time-point comparisons evaluated via Wilcoxon post hoc tests; for clarity, only significant results and trends are illustrated. Significant results and trends are presented with their corresponding *p*-values, which were derived from Wilcoxon post hoc tests performed to evaluate differences in changes in scores between the measurement points. Sample size for individual time points may vary slightly due to workshop attendance and data completeness (Available case analysis: BL n = 18; WS1 n = 18; WS2 n = 18; WS3 n = 15; WS4 n = 13).

**Figure 3:**
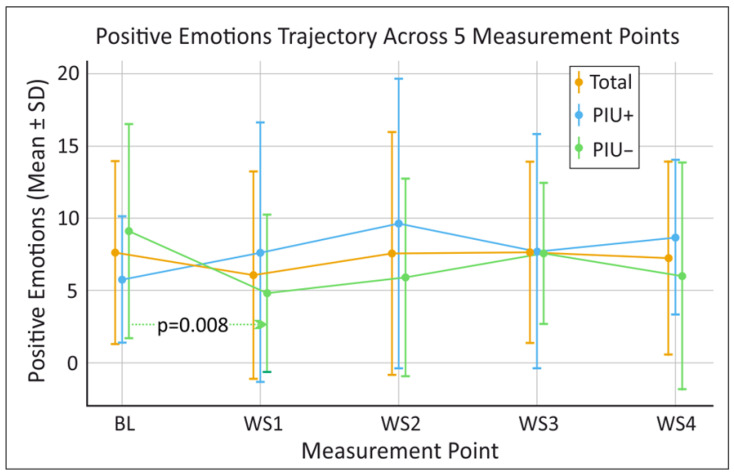
Longitudinal mean findings for positive emotions as measured by the Positive and Negative Affect Schedule (PANAS) over time for the total sample as well as for participants with and without problematic internet use (PIU+ and PIU−, respectively). BL = baseline, WS1 = workshop 1, WS2 = workshop 2, WS3 = workshop 3, WS4 = workshop 4. Horizontal dotted arrows indicate the time-point comparisons evaluated via Wilcoxon post hoc tests; for clarity, only significant results and trends are illustrated. Significant results and trends are presented with their corresponding *p*-values, which were derived from Wilcoxon post hoc tests performed to evaluate differences in changes in scores between the measurement points. Sample size for individual time points may vary slightly due to workshop attendance and data completeness (Available case analysis: BL n = 18; WS1 n = 18; WS2 n = 18; WS3 n = 16; WS4 n = 13).

Given the exploratory nature of the study and the small sample size, non-parametric Friedman tests were performed to test for differences in our outcome variables of positive emotions, negative emotions, and self-esteem as measured by the PANAS and SEKJ on repeated observations (BL, WS1, WS2, WS3, WS4) (see Figure 1, Figure 2 and Figure 3). In the overall sample no significant differences across the five time points (BL, WS1, WS2, WS3, WS4) were found for positive emotions (χ^2^(4) = 9.060, *p* = 0.060, W = 0.174), negative emotions (χ^2^(4) = 7.635, *p* = 0.106, W = 0.159), or self-esteem (χ^2^(4) = 5.528, *p* = 0.237, W = 0.092).

When analyses were conducted separately for the PIU+ subgroup, no significant time effects emerged for positive emotions (χ^2^(4) = 7.780, *p* = 0.100, W = 0.324), negative emotions (χ^2^(4) = 2.748, *p* = 0.601, W = 0.114), or self-esteem (χ^2^(4) = 5.720, *p* = 0.221, W = 0.204). Similarly, in the PIU− subgroup, the Friedman test revealed no significant temporal changes in positive emotions (χ^2^ (4) = 6.112, *p* = 0.191, W = 0.218), negative emotions (χ^2^ (4) = 8.774, *p* = 0.067, W = 0.366), or self-esteem (χ^2^ (4) = 2.316, *p* = 0.678, W = 0.072).

Wilcoxon post hoc tests were conducted for positive emotions, negative emotions and self-esteem between base line (BL) and all WS (BL–WS1, BL–WS2, BL–WS3 and BL–WS4), as well as between successive WS (WS1–WS2, WS2–WS3 and WS3–WS4) (see Figure 1, Figure 2 and Figure 3).

In the overall sample a significant decrease in negative emotions from BL (7.00 ± 7.16) to WS3 (5.07 ± 4.92) (*p* = 0.023) (see Figure 2). Additionally, a trend towards increased self-esteem was detected from BL (24.61 ± 10.15) to WS2 (26.94 ± 11.84) (*p* = 0.050) (see Figure 1). When analyses were performed separately for PIU+, no significant changes in negative and positive emotions were detected. However, trend-level changes were observed between WS3 (5.71 ± 4.72) and WS4 (8.50 ± 5.32) (*p* = 0.058), as well as between BL (9.13 ± 9.17) and WS3 (5.71 ± 4.72) (*p* = 0.063) for negative emotions (see Figure 2). In contrast, significant improvements in self-esteem were identified for PIU+ between BL (18.38 ± 7.54) to WS2 (21.75 ± 10.14) (*p* = 0.043), and from BL (18.38 ± 7.54) to WS3 (21.38 ± 9.07) (*p* = 0.042) were detected (see Figure 1).

For the PIU− group, positive emotions decreased significantly from BL (9.10 ± 7.42) to WS1 (4.80 ± 5.45) (*p* = 0.008) (see Figure 3), Negative emotions decreased from BL (5.30 ± 4.92) to WS1 (1.60 ± 2.17) (*p* = 0.007) and significantly increase from WS1 (1.60 ± 2.17) to WS2 (3.70 ± 4.95) (*p* = 0.027) (see Figure 2).

### 3.3. Narrative Feedback Evaluation Across All Workshops for Subgroups PIU+ vs. PIU−

Across WS, a shift in the distribution of reported categories was observed. Early WS (WS1 and WS2) were characterized by more negatively or ambivalently connoted experiences, whereas later workshops (WS3 and WS4) tended to show more frequent reports of neutral and positive categories (e.g., “okay,” “joy”). This overall pattern was evident in both groups.

**Figure 4:**
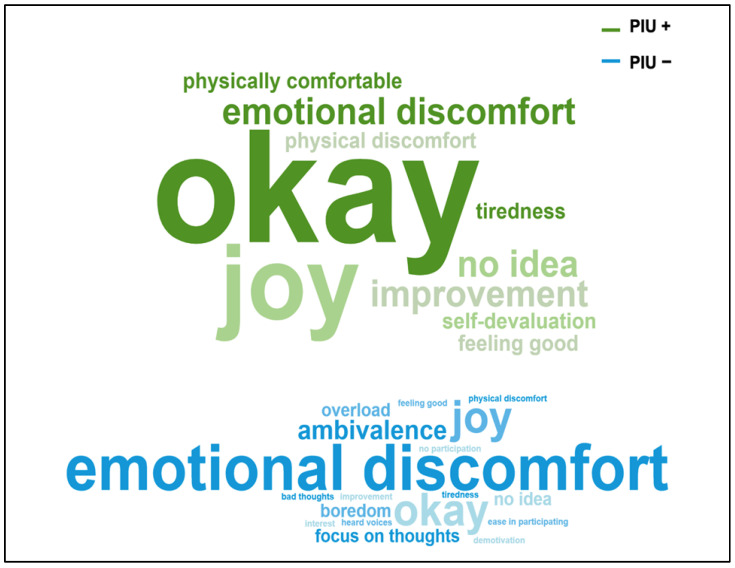
Word clouds of responses to question 1 (What thoughts and physical impressions did you have during the workshop? How did you feel during the dance exercises?) across all workshops (WS1–WS4) for the problematic internet use (PIU+) and the non-problematic internet use (PIU−) subgroups.

In WS1, the PIU+ group provided slightly more neutral and positive feedback and less negatively connoted feedback than the PIU− group. In WS2, the PIU+ group showed a small descriptive increase in neutral and positive feedback, alongside fewer negatively connoted categories. In WS3, the PIU− group reported slightly more neutral and positive feedback, yet also tended to report more negative emotions than the PIU+ group. In WS4, the PIU+ group reported slightly more positive emotions, with no negatively connoted feedback observed. Overall, across the course of the intervention, a descriptive trend emerged toward more frequent neutral and positive feedback and fewer negatively connoted responses. In the PIU+ group, this shift appeared descriptively more pronounced, with neutral categories reported more frequently and negatively connoted experiences declining in later workshops (see Figure 4). For detailed category frequencies by session, as well as total frequencies across all workshops divided by group, see Appendix A Table A2.

## 4. Discussion

The present dance intervention aimed to address key affective symptoms frequently observed in adolescent psychiatric populations—namely elevated negative emotions (Aldao et al., 2016) and low self-esteem (Henriksen et al., 2017)—while simultaneously promoting positive emotions, a known contributor to resilience and psychological well-being (Gloria & Steinhardt, 2016). Dance programs that combine structured movement with rhythmic and expressive elements provide a promising mean of reducing negative emotions and enhancing positive emotional experiences, while also enhancing self-esteem (Duberg et al., 2016; Koch et al., 2019; Schwender et al., 2018; Zhang & Wei, 2024). Given the high prevalence of PIU in clinical youth populations (Fuchs et al., 2018; Winds et al., 2022), and its association with under-recognition in inpatient settings (Pei-Chen Chang & Hung, 2012) as well as challenges related to smartphone restrictions during treatment (Burke et al., 2022), it is clinically relevant to examine whether adolescents with PIU+ and also PIU− benefit similarly from such emotion-focused dance movement interventions.

### 4.1. The Impact of PIU on Clinical Presentation and Outcome Variables

In our sample, adolescents with PIU+ reported significantly lower self-esteem and a higher burden of internalizing symptoms than their PIU− peers. These findings are consistent with the broader literature, which has repeatedly shown associations between PIU, low self-esteem, and internalizing psychopathology in adolescents (Villanueva-Blasco et al., 2026). PIU, particularly social media use, may foster social comparisons that increase dissatisfaction with one’s life and subsequently reduce self-esteem or result in depression, especially among adolescents (Muris & Otgaar, 2023). However, as noted in previous research, the directionality of these associations remains unclear: low self-esteem and internalizing symptoms may act as predisposing vulnerabilities for the development of PIU, but they may also be amplified over time by maladaptive patterns of internet use (Villanueva-Blasco et al., 2026). Our cross-sectional baseline data therefore align with existing evidence but do not allow causal inference.

Our findings suggest that total internet use time did not differ significantly between the PIU+ and PIU− groups, which is consistent with our categorization strategy. Interestingly, the absence of a group difference in screen time underscores that the defining feature of PIU in this sample was not usage duration per se, but rather compulsive and dysregulated patterns of use, as captured by the CIUS. This distinction aligns with recent meta-analytic evidence indicating that total screen time is only a weak predictor of health and educational outcomes (r = −0.14 to 0.33), whereas qualitative and psychological characteristics of use appear to be more consequential (Sanders et al., 2024). From a clinical perspective, conceptualizing PIU as a construct characterized by loss of control rather than quantity of use may help explain differential treatment responses and may be relevant for tailoring future interventions.

### 4.2. Self-Esteem in the Context of the Dance Intervention

Self-esteem can be conceptualized as an individual’s appraisal of their abilities in relation to social norms and expectations. It refers to how individuals perceive and evaluate themselves either globally or within specific life domains (Rosenberg et al., 1995), such as academic performance and social functioning (Eisenberg & Schneider, 2007; Lysaker et al., 2007). While early theoretical accounts described self-esteem as a unitary, general attitude toward the self (Maslow, 1954; Rosenberg, 2006), more recent frameworks emphasize its multidimensional nature, comprising evaluations across distinct domains of functioning (Cast & Burke, 2002). Moreover, self-esteem can be understood in terms of cognitive appraisal of the self (Ek et al., 2008), emotional aspects related to self-acceptance (Brown & Marshall, 2006), or an integration of both components (Tafarodi & Swann, 1995, 2001). Across theoretical perspectives, self-esteem is consistently linked to general psychological well-being (Du et al., 2017). Low self-esteem is a known risk factor for mental health difficulties and is a central treatment target across psychotherapeutic modalities (Bos et al., 2006). Movement-based interventions, including dance, have been linked to improvements in self-esteem and embodied self-experience (Duberg et al., 2016; Moula et al., 2022; Schwender et al., 2018).

In line with this, our results revealed a trend towards an increase in self-esteem from BL to WS 2 in the total sample. When dividing the cohort by PIU status, however, this improvement was predominantly driven by the PIU+ group, which showed significant gains at multiple time points. One possible explanation is that adolescents with PIU+ tend to restrict real-life experiential domains because online activities dominate their reward cycles (Kuss & Lopez-Fernandez, 2016). Consequently, they may have fewer opportunities to experience competence or mastery offline. Participating in a dance activity may therefore have offered these adolescents a broader range of embodied experiences and successful task engagement, supporting a sense of competence and self-efficacy—processes essential to self-esteem development. Indeed, earlier studies and broader reviews indicate that physical and dance-based activities may reduce symptoms of internet addiction in adolescents, with self-esteem and self-efficacy emerging as potential mediating mechanism (Jia et al., 2025; Wang et al., 2025).

### 4.3. Positive Emotions in the Context of the Dance Intervention

Positive emotions are closely intertwined with resilience, contribute to overall well-being (Gloria & Steinhardt, 2016), and play an important role in broadening cognitive and emotional perspectives during treatment (Fredrickson, 2001). In the present study, the only significant result was a decline in positive emotions from BL to WS1 in the PIU− group; no further significant changes were detected across the intervention overall or between time points. Nevertheless, the graphical representation suggests an interesting pattern, indicating diverging temporal dynamics in emotional responses between adolescents with PIU+ and PIU−.

In the PIU− group, positive emotions declined significantly from BL to WS1 and remained lower than at BL levels throughout the intervention. This may reflect a critical or evaluative stance towards the intervention often observed in clinically burdened adolescents with internalizing symptoms (Birnkammer & Calvano, 2023; Grebosz-Haring & Thun-Hohenstein, 2024).

In contrast, adolescents with PIU+ exhibited a continuous—albeit non-significant—increase in positive emotions across workshops. This graphical trend was descriptively mirrored in the narrative feedback: while the PIU− group initially reported more ambivalent responses, the PIU+ group more frequently reported neutral and positive categories (e.g., “okay” and “joy”), particularly in the final workshop.

Hypothetically, the intervention may have been perceived increasingly positively by adolescents with PIU+. One possible explanation is that, for adolescents with PIU+, dance may have represented a novel, non-internet-based form of activation that nevertheless resonates with familiar digital practices, such as dance-related content on social media platforms (Montag et al., 2021; Roth et al., 2021). The active and performative elements of the intervention may have facilitated engagement and contributed to incremental increases in positive emotional experiences. These observations are consistent with previous findings on arts- and exercise-based interventions, which have been associated with reductions in internalizing symptoms and improvements in emotional well-being among adolescents with psychological burden or PIU (Jung & Park, 2025; Wang et al., 2025).

### 4.4. Negative Emotions in the Context of the Dance Intervention

Negative emotions and difficulties in emotion regulation are central features of many psychiatric disorders and are typically reported by patients as major sources of subjective distress (Bradley et al., 2011; Mesurado et al., 2018). In our sample, adolescents with PIU+ consistently exhibited higher levels of negative emotions across all measurement points. This observation aligns with the Interaction of Person-Affect-Cognition-Execution (I-PACE) model (Brand et al., 2016, 2019). According to this framework, problematic internet use is not merely a behavioral addiction but the result of a complex interaction between predisposing factors (e.g., psychopathology, personality traits) and affective/cognitive responses to specific triggers (Brand et al., 2016). In the context of our study, the elevated negative emotions observed in the PIU+ group may represent a ‘predisposing affective state’ that drives adolescents towards the maladaptive use of digital media as a strategy for mood regulation. According to the I-PACE model, while internet use may initially serve as a coping mechanism to alleviate distress, long-term loss of control can lead to the reinforcement of negative emotional states when offline (Brand et al., 2019). This theoretical foundation assists in clarifying why the PIU+ group in our investigation began with a higher emotional load and exhibited a more incremental, ‘stepped’ enhancement during the dance intervention, as they were required to shift from digital coping to a physical, embodied expression of emotion regulation.

Consistent with previous findings (Li et al., 2023; Moula et al., 2022), we observed a significant reduction in negative emotions from BL to WS 3 in the overall sample. In the PIU+ group, this reduction did not reach statistical significance; graphically, negative emotions showed a clear downward trend. While these changes occurred during the intervention period, it suggests that the dance workshops were associated with a temporary reduction in emotional distress, although contribution from other therapeutic factors within the inpatient setting cannot be ruled out (Li et al., 2023). The PIU− group exhibited a significant decline in negative emotions at the start of the intervention, the PIU+ group showed a more gradual improvement up to WS 3. This pattern might be linked to the sustained motor activation provided by the workshops, potentially influencing affective functioning in adolescents with PIU+, who generally tend to engage in lower levels of physical activity (Shristi et al., 2024; Zhu et al., 2025). In general reduction in negative affect in PUI+ is desirable, particularly given the heightened emotional vulnerability that characterizes this population (Villanueva-Blasco et al., 2026). Although negative affect in the total sample and PIU+ declined during the active phase of the dance intervention it increased again toward the end. This pattern may reflect a termination effect reported in psychotherapy literature (Rabinowitz et al., 2025), as the conclusion of a structured, supportive group can elicit separation-related distress, loss of routine, and anticipatory stress, which are known to transiently increase negative emotions in adolescent group and psychotherapy settings. Finally, meta-analytic evidence indicates that while dance and dance movement–based interventions are generally associated with reduced negative affect, emotional trajectories can be heterogeneous, making end-of-intervention fluctuations plausible in clinically burdened adolescent populations (Koch et al., 2019).

### 4.5. Limitations and Strength of the Study

A major limitation of the study is the small sample size and the absence of a control or comparison condition. Consequently, it is difficult to determine whether the observed changes were attributable to the dance intervention specifically or to non-specific factors, such as the social attention within the group, the novelty of the activity, or the passage of time during inpatient treatment. While such designs are common in pilot trials to assess feasibility and initial trends, the lack of a control group, combined with the short intervention duration and absence of follow-up data, limits our ability to draw firm conclusions regarding the stability and specificity of the effects. Furthermore, the classification into PIU+ and PIU− categories was based on a validated CIUS cut-off score of 28. Although this threshold is well-established in clinical research (Guertler et al., 2014; Rehbein et al., 2025; Rumpf et al., 2014; Wartberg et al., 2014), we acknowledge the ongoing debate surrounding the dimensional nature of PIU as a dimensional construct.

Given the small sample size, a more stringent cut-off or a dimensional analysis was not feasible without further compromising statistical power. It remains possible that a higher threshold or dimensional approach in a larger sample would yield clearer differences between groups. However, the absence of significant differences in total screen time between the PIU+ and PIU− groups is consistent with recent findings (Sanders et al., 2024), suggesting that the compulsive nature of internet use rather than mere duration may constitute the primary driver of psychological distress.

Another limitation concerns the post hoc comparisons. No alpha-level adjustments (e.g., Holm-Bonferroni) were made in order to preserve statistical power in this small-scale exploratory pilot study. This approach increases the risk of Type I errors (false-positives); therefore, the reported *p*-values should be interpreted as indicative of potential trends rather than definitive evidence. In addition, graphical findings that did not reach statistical significance were reported. This decision was made to facilitate hypothesis generation and to explore preliminary patterns within the framework of a pilot study.

Taken together these limitations underscore the need for cautious interpretation and constrain the strength of the conclusions. This consideration aligns with previous critiques of dance-based activities in treatment settings (Grebosz-Haring et al., 2023). Further research should employ more rigorous, large-scale designs to determine under which conditions dance interventions reliably produce emotional benefits and for whom.

A strength of our study is that it resonates with previous work demonstrating that arts engagement can bolster youth mental health and resilience (Golden et al., 2024). Specifically, dance and sport interventions may lead to a reduction in emotional distress in youth with mental health challenges (Moula et al., 2022). Furthermore, to our knowledge, our study was the first to examine the impact of PIU on emotions and self-esteem during a dance intervention within a child and adolescent psychiatric sample. The study was conducted in a clinical setting with adolescents presenting with psychopathology, which increases its clinical relevance and ecological validity. In this study, an observational analytic approach was employed. Importantly, both the study team and the dance instructor were blinded to participants’ PIU status, thereby minimizing the risk of unconscious bias in the implementation and facilitation of the workshops. This blinding reduced the likelihood that differential support or altered instructional approaches were provided to adolescents with pathological internet use, helping to ensure a consistent intervention delivery across participants.

## 5. Conclusions

The present pilot study provides preliminary indications that a dance-based intervention in a child and adolescent psychiatric setting may coincide with positive developments in emotional experience and self-esteem, particularly among youths with elevated levels of PIU. Although exploratory, these findings point to the potential importance of assessing PIU in clinical child and adolescent populations, as higher levels of PIU may be associated with distinct patterns of emotional functioning and self-related evaluations. Early identification of PIU could therefore enrich the clinical formulation and inform contribute treatment planning.

Adolescents with higher PIU levels showed descriptive tendencies toward positive engagements with the dance workshops. From a clinical perspective, this suggests that movement-based and body-oriented activities might constitute a feasible, low-threshold means of fostering therapeutic participation, even in the absence of PIU-specific treatment programs in routine care. By offering opportunities for positive movement experiences, such interventions may support perceived self-competence and self-esteem, which could in turn enhance overall readiness to engage in treatment.

Taken together, these exploratory results tentatively highlight dance- and movement-based approaches as a potentially useful adjunct within child and adolescent psychiatric settings, particularly for adolescents with clinically relevant PIU. However, given the small sample size, short intervention duration, and lack of a control group, the present findings are hypothesis-generating and do not allow for causal inferences. Further methodologically rigorous and larger-scale studies are needed to determine the stability, specificity, and clinical relevance of these preliminary observations.

## Figures and Tables

**Table 1:** Descriptive Statistics for Age, Internet Use, Internalizing and Externalizing Symptoms, positive and negative emotions (PANAS), self-esteem (SEKJ) for total sample and PIU+ and PIU− subgroups.

	Total Sample (*n* = 18)	PIU+ (*n* = 8)	PIU− (*n* = 10)	Test ^1,2^	Effect Size ^3,4^
Demographics					
Age (m, SD)	15.39 (1.50)	15.88 (0.99)	15.00 (1.76)	*p* = 0.140 ^1^	0.348 ^4^
Weekday internet use (m, SD)	6.23 (3.79)	6.50 (3.12)	6.10 (4.41)	*p* = 0.501 ^1^	0.159 ^4^
Weekend internet use (m, SD)	8.11 (5.46)	8.75 (3.69)	7.60 (6.70)	*p* = 0.105 ^1^	0.382 ^4^
Internalizing Symptoms (m, SD)	1.34 (0.79)	1.80 (0.52)	0.97 (0.79)	***p* = 0.041 ^1^**	0.482 ^4^
Pathological (n, %)	12 (66.7)	8 (100.0)	4 (40.0)	***p* = 0.013 ^2^**	0.632 ^3^
Externalizing Symptoms (m, SD)	0.81 (0.70)	0.57 (0.27)	1.00 (0.88)	*p* = 0.656 ^1^	0.662 ^4^
Pathological (n, %)	4 (22.2)	1 (12.5)	3 (30.0)	*p* = 0.588 ^2^	0.209 ^3^
PANAS PE (BL) (m, SD)	7.61 (6.34)	5.75 (4.46)	9.10 (7.41)	*p* = 0.449 ^1^	0.178 ^4^
PANAS NE (BL) (m, SD)	7.00 (7.16)	9.13 (9.17)	5.30 (4.92)	*p* = 0.284 ^1^	0.253 ^4^
SEKJ (BL) (m, SD)	24.61 (10.15)	18.37 (7.54)	29.60 (9.38)	***p* = 0.018 ^1^**	0.556 ^4^
SEKJ below norm (n, %)	10 (55.6)	7 (87.5)	3 (30.0)	***p* = 0.025 ^2^**	0.575 ^3^

Note: According to the data, tests between PIU groups were performed. ^1^ Mann–Whitney U-tests for continuous measures and ^2^ Fishers Exact Test for categorical measures; PIU, problematic use of the internet; m, mean; SD, standard deviation; BL, baseline; PANAS, Positive and Negative Affect Schedule; PE, positive emotions; NE, negative emotions; SEKJ, Self-Esteem Inventory for Children and Adolescents; ^3^ Cramer’s V, interpretation according to Cohen (1988) 0.10 (small effect), 0.30 (medium effect), and 0.50 (large effect), ^4^ r, interpretation according to Cohen (1988): 0.10 (small effect), 0.30 (medium effect), and 0.50 (large effect). Values in bold indicate statistical significance (*p* < 0.05).

## Data Availability

The data presented in this study are available on request from the corresponding author due to legal reasons in clinical populations.

## Abbreviations

The following abbreviations are used in this manuscript:

BL	baseline
CIUS	compulsive internet use scale
DISYPS-III SBB-Screen	self-rating scale of the German Diagnostic System for Mental Disorders, according to the ICD-10 and DSM-5 criteria for children and adolescents (in German: Diagnosesystem für psychische Störungen nach ICD-10 und DSM-5 für Kinder und Jugendliche)
PANAS	positive and negative affect schedule
PIU	problematic internet use
PIU+	participants with problematic internet use
PIU−	participants without problematic internet use
Scheme	Self-esteem inventory for children and adolescents (German: Selbstwertinventar für Kin-der und Jugendliche)
WS	workshop

## Appendix A

**Table A1:** Missing data for PANAS positive and negative affect and SEK-J, divided by PIU− and PIU+ groups.

Variables	PIU−	PIU+
PE_WS1	0	0
NE-_WS1	0	0
PE_WS2	0	0
NE-_WS2	0	0
PE_WS3	1	1
NE_WS3	2	1
PE_WS4	3	2
NE_WS4	3	2
SE_WS1	0	0
SE_WS2	0	0
SE_WS3	0	0
SE_WS4	2	1

Note. PE, positive emotions; NE, negative emotions, assessed with PANAS, Positive and Negative Affect Schedule; SE, self-esteem, assessed with SEKJ, Self-Esteem Inventory for Children and Adolescents.

**Table A2:** Frequency of responses by category and session, divided by PIU− and PIU+ groups.

Categories	WS 1_PUI−	WS 1_PUI+	WS 2_PUI−	WS 2_PUI+	WS 3_PUI−	WS 3_PUI+	WS 4_PUI−	WS4_PUI+	Total_PUI−	Total_PUI+
overload	2	0	0	0	1	0	0	0	3	0
emotional discomfort	2	1	3	1	0	1	1	0	6	3
demotivation	1	0	0	0	0	0	0	0	1	0
physical discomfort	0	1	0	0	1	0	0	0	1	1
self-devaluation	0	1	0	0	0	0	0	0	0	1
heard voices	0	0	1	0	0	0	0	0	1	0
bad thoughts	0	0	0	0	0	0	1	0	1	0
focus on thoughts	0	0	0	0	0	0	2	0	2	0
ambivalence	1	0	1	0	1	0	0	0	3	0
tiredness	0	0	1	1	0	0	0	0	1	1
boredom	1	0	1	0	0	0	0	0	2	0
no participation	0	0	0	0	1	0	0	0	1	0
okay	0	2	2	2	1	3	2	3	5	10
no idea	1	1	1	0	0	0	0	0	2	1
interest	1	0	0	0	0	0	0	0	1	0
improvement	0	0	1	2	0	0	0	0	1	2
physically comfortable	0	0	0	1	0	0	0	0	0	1
feeling good	0	0	0	0	1	0	0	1	1	1
ease in participation	0	0	0	0	0	0	1	0	1	0
joy	1	1	0	3	4	1	0	3	5	8
MISSINGS	2	1	2	1	3	3	3	2		
